# Prevalence of deleterious *ATM* germline mutations in gastric cancer patients

**DOI:** 10.18632/oncotarget.5944

**Published:** 2015-10-23

**Authors:** Dong-Sheng Huang, Hou-Quan Tao, Xu-Jun He, Ming Long, Sheng Yu, Ying-Jie Xia, Zhang Wei, Zikai Xiong, Sian Jones, Yiping He, Hai Yan, Xiaoyue Wang

**Affiliations:** ^1^ Department of Surgery, Zhejiang Provincial People's Hospital, Hangzhou, Zhejiang, China; ^2^ Key Laboratory of Gastroenterology of Zhejiang Province, Hangzhou, Zhejiang, China; ^3^ Department of Surgery, No.2 Hospital of Deyang City, Deyang, Sichuan, China; ^4^ Genetron Health (Beijing) Technology, Co. Ltd., Changping, Beijing, China; ^5^ Personal Genome Diagnostics, Baltimore, MD, USA; ^6^ The Preston Robert Tisch Brain Tumor Center at Duke, and Department of Pathology, Duke University Medical Center, Durham, NC, USA; ^7^ State Key Laboratory of Medical Molecular Biology, Institute of Basic Medical Sciences, Chinese Academy of Medical Sciences, Peking Union Medical College, Beijing, China

**Keywords:** familial gastric cancer, ATM, cancer susceptibility, hereditary cancer gene panel

## Abstract

Besides *CDH1*, few hereditary gastric cancer predisposition genes have been previously reported. In this study, we discovered two germline *ATM* mutations (p.Y1203fs and p.N1223S) in a Chinese family with a history of gastric cancer by screening 83 cancer susceptibility genes. Using a published exome sequencing dataset, we found deleterious germline mutations of *ATM* in 2.7% of 335 gastric cancer patients of different ethnic origins. The frequency of deleterious *ATM* mutations in gastric cancer patients is significantly higher than that in general population (p=0.0000435), suggesting an association of *ATM* mutations with gastric cancer predisposition. We also observed biallelic inactivation of *ATM* in tumors of two gastric cancer patients. Further evaluation of *ATM* mutations in hereditary gastric cancer will facilitate genetic testing and risk assessment.

## INTRODUCTION

Gastric cancer is the third most common cause of cancer mortality worldwide, accounting for 723,000 deaths in 2012 [1]. Familial aggregation of gastric cancer is common in about 10% of the cases [2]. Although environmental risk factors, such as chronic infections by *Helicobacter pylori* and Epstein-Barr virus, partly explain the familial clustering of gastric cancer [3, 4], genetic susceptibility also has a role in the disease [5]. Germline mutations in *CDH1* genes contribute to about 40% of hereditary diffuse gastric cancer (HDGC) cases [6], and Lynch syndrome families with inherited mutations in the mismatch repair genes are at an increased risk for gastric cancer [7-9]. Recently, germline mutations in *MAP3K6* were also found to be associated with familial gastric cancer [10]. However, for most gastric cancer cases, whether genetic events contribute to cancer susceptibility remains unknown.

Here we reported a Chinese family from Sichuan China with a history of familial gastric cancer. We sequenced the germline DNAs from four of the family members and identified two rare mutations in ataxia telangiectasia mutated (*ATM*) genes, one of them resulting in a truncated protein. Using a TCGA dataset of 335 gastric cancer patients, we estimated the prevalence of *ATM* germline mutations and identified 9 deleterious mutations including 6 truncating mutations.

## RESULTS

### ATM mutations in a familial gastric cancer family

**Figure 1:**
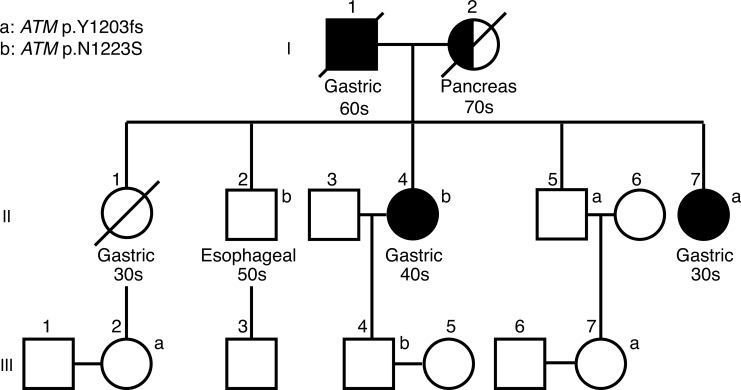
Pedigree of the Sichuan Chinese family Individuals with gastric cancers are shaded in black. Half-shaded symbols indicate individuals with non-gastric cancers. Generation I-III are indicated. All the members of the family, excluding those who were deceased, were tested for two *ATM* mutations (ATM p.Y1203fs and p.N1223S). Age of initial diagnosis was indicated as 30s, 40s, 50s, 60s and 70s. Carriers for these two ATM mutations are indicated by a and b, respectively.

In routine clinical practices at Zhejiang Provincial People's Hospital, we encountered a Chinese family from Sichuan province with a history of familial gastric cancer (Figure 1). The family has four members with gastric cancer, and two of them were diagnosed under age 50.

The proband, affected individual II-7, was diagnosed with highly differentiated gastric adenocarcinoma at age 39 and underwent a partial gastrectomy. Pathological examination revealed invasion into superficial layer of muscularis propria.

The proband's older sister, individual II-4, was diagnosed with metastatic gastric carcinoma at age 41 and underwent a partial gastrectomy. A stomach biopsy of this patient revealed a moderately to poorly differentiated stomach adenocarcinoma with invasion into superficial layer of muscularis propria. Individual II-4 was tested negative for *H. pylori* infection.

The proband's eldest brother, individual II-2, was reported to have esophageal squamous cell carcinoma and underwent a partial esophageal resection at age 53. A re-examination of the biopsy showed esophageal squamous epithelial atypical hyperplasia with canceration. Another brother of the proband, individual II-5, is unaffected at age 54. Little information was available for the proband's eldest sister (individual II-1) because she deceased at age 38 with gastric cancer.

The proband's father (individual I-1) died of gastric cancer at age 68. The proband's mother (individual I-2) died of pancreatic cancer at 79. All the individuals in the third generation are between 26 and 32 years old and unaffected.

To identify hereditary factors for gastric cancer in this family, we sequenced genomic DNAs from individuals II-2, II-4, II-5 and II-7 using a custom panel-based assay. The custom panel contained 83 genes previously suggested to be associated with risk for hereditary cancer, including *CDH1* and five Lynch syndrome genes (*EPCAM, MLH1, MSH2, MSH6, PMS2;* see Supplementary Table 1 for the list of 83 genes).

We identified an average of 80.5 variants per individual in 83 genes (Supplementary Table 2). No mutations in *CDH1* genes or mismatch genes were found. We prioritized the variants using the population frequency information from the 1000 Genomes Project [11], assuming the variants with minor allele frequency (MAF) greater than 0.01 to be benign or low penetrance. In total, we found 22 rare variants with MAF less than 0.01 or not reported in 1000 genomes database, none of which segregated with the gastric cancer in this family. Of the total 22 variants, the most interesting one was a frameshift deletion in *ATM* (NM_000051.3:c.3609delT, p.Y1203fs). Although it was not shared among all the affected individuals (found in II-5 and II-7), it was the most damaging mutation in all the genes tested. Interestingly, another rare non-synonymous mutation in ATM (c.3668A > G, p. N1223S) was identified in individuals II-2 and II-4, which did not co-occur with ATM Y1203fs in any of the four individuals.

Neither of the two *ATM* mutations was reported before. They are probably inherited from the parents of the proband, although we did not have their genomic DNAs to validate. To validate our findings, we sequenced the exon 25 of *ATM* in 12 members of the Sichuan family (individuals II-2, II-3, II-4, II-5, II-7 and individuals III-1 to III-7) using Sanger sequencing (Supplementary Figure 1). Individual III-2, one niece of the proband, had the p.Y1203fs mutation. She is in her 20s and unaffected. It is possible that her mother, the deceased patient (individual II-1), was also a carrier of p.Y1203fs. None of the spousal controls had mutations in *ATM*.

### Prevalence of deleterious ATM germline mutations in gastric cancer patients

**Figure 2:**
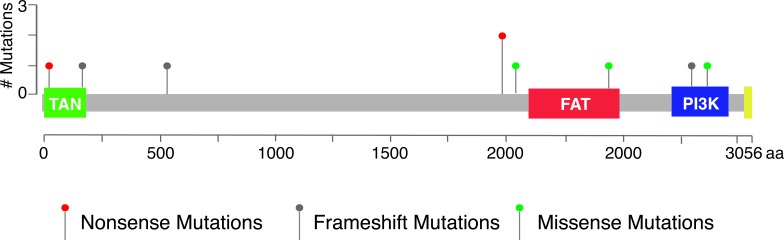
Distribution of nine deleterious mutations in TCGA dataset in relation to the predicted functional domains of *ATM* Red: Nonsense mutations; Green: Missense mutations; Grey: Frameshift mutations.

*ATM* is a well-known tumor suppressor but not a known predisposition gene for gastric cancer. The discovery of two rare *ATM* mutations (one is likely to be deleterious) in the family promoted us to investigate the frequency of deleterious *ATM* germline mutations in gastric cancer patients. We analyzed 335 cases in the stomach adenocarcinoma study reported by The Cancer Genome Atlas (TCGA) Research Network [12]. From the sequencing data of normal blood DNA of the 335 patients, we identified 3 different germline heterozygous frameshift deletions and 2 nonsense mutations in *ATM*, with one nonsense mutation (p.E1978X) occurred in two patients (Table 1 and Figure 2). We did not have any information on the relativeness of these two patients. The p.E1978X mutation was reported in ataxia-telangiectasia (AT) families before [13]. In addition, three rare nonsynonymous mutations may be damaging to the functions of ATM protein, as they were previously reported in AT patients (Table 1). The three missense mutations were considered deleterious by at least two *in silico* prediction methods (Supplementary Table 3). One of the three missense mutations, p.V2424G (c.7271T > G), was shown to be associated with increased risk of breast cancer in AT families [14]. Another missense mutations, p.R2032K (c.6095G > A) was also reported in familial pancreatic cancer families [15].

**Table 1:** Summary of heterozygous deleterious ATM variants found in patients with gastric cancer

Nucleotide (genomic)	Nucleotide (cDNA)	Amino Acid	Type	Age at Onset	Number of affected	TCGA_ID	Previous Reported
11:108098418C>T	c.G67T	p.R23X	Nonsense	71	1	TCGA-RD-A8MV*	NA
11:108106511delTTCT	c.446_449del	p.I149fs	Frameshift deletion	46	1	TCGA-BR-6564	NA
11:108121753delAG	c.1561_1562del	p.R521fs	Frameshift deletion	NA	1	TCGA-HF-7133	NA
11:108183151G>T	c.G5932T	p.E1978X	Nonsense	41,64	2	TCGA-BR-6710 TCGA-BR-8077	Li and Swift et al., 2000
11:108214065delTTT CAGTGCC	c.8385_8395del	p.D2795fs	Frameshift deletion	57	1	TCGA-VQ-AA6F	NA
11:108216616T>G	c.8565T>G	p.S2855R	Missense	64	1	TCGA-BR-7196	Castellvi-Bel et al., 1999
11:108186638G>A	c.6095G>A	p.R2032K	Missense	70	1	TCGA-BR-8365	Li and Swift et al., 2000
11:108199929T>G	c.7271T>G	p.V2424G	Missense	71	1	TCGA-RD-A7BW	Stankovic et al., 1998

* a somatic mutation (c.1024dupA, p.V341fs) was reported in the tumor DNA of the c.G67X carrier.

In total, 9 of the 335 patients were characterized as carriers of deleterious *ATM* mutations (Table 1 and Figure 2). To estimate the frequency of deleterious mutations in general population, we analyzed the variants reported in the phase 3 data of the 1000 genomes project. In 2054 individuals, we found four nonsense mutations, one splicing site mutation and one previously reported missense mutation in *ATM* (Supplementary Table 4). The frequency of *ATM* deleterious mutations was significantly higher in the gastric cancer patients than that in general population (9/335 for TCGA gastric cancer data vs. 6/2054 for 1000 genomes data; odds ratio = 9.41;the Fisher exact test, p = 0.0000435).

Among the 9 carriers of deleterious ATM mutations, two of them were diagnosed of gastric cancer before 50 years old, with the youngest age of onset at 41. The average age-of-initial diagnosis of these patients was 60.5, while the mean age-of-onset of all the noncarriers was 65.2. The difference was not statistically significant (Student's t test, p = 0.098).

### Biallelic inactivation of ATM in gastric cancer patients

In the tumor DNA of the germline *ATM* p.R23X (c.67G > T) carrier in the TCGA dataset, a somatic frameshift insertion in *ATM* (c.1024dupA, p.V341fs) was reported [12]. The co-occurrence of a germline mutation and a somatic mutation in the same patient is consistent with a “two-hit” model for tumor suppressor genes. Other mechanisms of inactivating both alleles of *ATM* may also exist, such as chromosomal level deletions and promoter methylation. The impurity of stomach tumor tissues precluded our search for loss-of-heterozygosity directly from TCGA sequencing data. However, copy number loss at *ATM* locus was found in three samples of *ATM* deleterious mutations carriers, according to TCGA's GISTIC analysis [12].

In search of other patients with germline mutation in *ATM*, we sequenced *ATM* genes in 20 additional patients in Zhejiang Renmin Hospital. We discovered a rare germline *ATM* mutation (rs55870064, c.4949A > G, p. N1650S) with minor allele frequency of 0.0024 in 1000 genomes project data in one patient. Sequencing DNA from his blood, tumor tissue and lymph node biopsy showed the complete loss of the reference allele and the retention of the mutant G allele in lymph node (Supplementary Figure 2). Loss of heterozygosity for *ATM* in this patient is also consistent with the tumor suppressor role of *ATM*. Interestingly, this patient's family members had esophageal squamous cell carcinoma and leukemia (Supplementary Figure 3).

## DISCUSSION

ATM is a kinase involved in cell cycle control and cancer development [16]. Biallelic inactivation of *ATM* results in Ataxia-telangiectasia [16, 17]. Germline mutations in *ATM* have also been associated with a moderately increased risk for breast cancer and pancreatic cancer [15, 18]. A predisposition role for *ATM* in gastric cancer was not known, although some evidence of excess risks was reported from Ataxia-telangiectasia family studies [19, 20].

In our study, we found a frameshift mutation (p.Y1203fs) and a missense mutation (p.N1223S) in *ATM* in a Chinese gastric cancer family. There are some limitations in our pedigree study to prevent us conclude the causal roles of either mutation. First, we only sequenced 83 selected genes and did not have information of mutations in other genes. Also, we only analyzed point mutations, small insertions and deletions thus may miss other types of mutations. Third, although the frameshift *ATM* mutation (p.Y1203fs) is likely to be deleterious, the individual II-5 who carry the mutation was not affected. It could be due to incomplete penetrance.

Nevertheless, the discovery of deleterious *ATM* mutations raises the question of the predisposition role of *ATM* in gastric cancer. By mining a public dataset, we found 2.7% of gastric patients of different ethnic origins have deleterious mutations in *ATM*, 2/3 of which would result in truncated forms of the encoded ATM proteins. In addition, we found the biallelic inactivation of *ATM* by both a germline and a somatic deleterious mutation in the tumor of one patient. These data supports an association of *ATM* mutations with gastric cancer susceptibility.

While we were preparing this manuscript, a GWAS study by *Helgason et al.* was published online, reporting the discovery of a gastric cancer association with loss-of-function mutations in *ATM* in a European population [21]. Our observation of a truncating mutation of *ATM* in a Chinese family of gastric cancer history, as well as the prevalence of deleterious *ATM* variants in patients of different ethnic origins are consistent with their results. Future work is needed to evaluate *ATM* mutations for risk assessment and therapy development in gastric cancer.

## MATERIALS AND METHODS

### Study participants

This study was approved by the Institutional Review Board of the Zhejiang Provincial People's Hospital. Study subjects include 12 members of a Sichuan Chinese family and one patient from Zhejiang, China. Informed consent was obtained from all individuals or their family members for the biospecimens and medical records used in this study. To protect the privacy of the patients, data used in this study will not be deposited in public databases. Data used in this study could be requested from the corresponding authors pending approval from the family.

### Genomic DNA preparation

Blood samples were obtained from 12 members of the SiChuan family under IRB approved protocols. Genomic DNA was extracted from blood using QIAamp DNA mini kit (cat# 51304, Qiagen, Valencia, CA, USA) or QIAquick FFPE DNA Kit (cat# 56404, Qiagen, Valencia, CA, USA) according to the manufacturer's protocols.

### Library preparation for targeted gene sequencing

A total of 1μg DNA was used to generate genomic DNA libraries according to the protocols suggested by Illumina. A custom targeted capture kit was designed using Agilent Sureselect tools, covering all exons of the 83 genes (Supplementary Table 1). The total coverage of the chip was 434 kilobases. The target enrichment was performed using the Agilent SureSelect Target Enrichment kit (Agilent Technologies). The amplified DNA libraries were sequenced with the Illumina MiSeq Genome Analyzer (Illumina, San Diego, CA, USA), yielding 75 bp of paired end sequences.

Sequenced reads were then aligned to the reference human genome (GRCh37) using Burrows-Wheeler Aligner (BWA, v.0.7.10), and variant calls were generated by the Genome Analysis Toolkit (GATK, v 2.3.1). Sequencing statistics for each sample were listed in Supplementary Table 2. The coding regions of the 83 genes were selected for their association with cancer risk based on published evidences. Mutations were filtered for sequencing quality and depth of coverage.

### Validation of ATM mutations by Sanger Sequencing

Selected regions of ATM genes were amplified from genomic DNA by PCR, using primers TGGTTCGTGCAGGTTTTAGAG and TGGTATGTGTGTTGCTGGTG for c.3609delT and c.3668A > G, as well as primers GTTCAGATTCATTCCCTAC and GGCAACAGAAAACATACA for c.4949A > G. Amplified fragments were sequenced using Sanger fluorescent sequencing. Sequencing tracers were analyzed using Mutation-Surveyor (Soft Genetics, State College, PA, USA).

### TCGA gastric cancer dataset

Germline variants information for 335 gastric cancer patients was downloaded through TCGA data portal on Feb. 26^th^, 2015. Raw sequences in BAM format for 9 patients with deleterious germline mutations in *ATM* were downloaded from CGHub (https://cghub.ucsc.edu/). The access to these controlled-access data was approved by TCGA data access committee in Jan. 2015.

### Variants characterization

Variants were annotated using annovar [22]. Online databases including the Human Gene Mutation Database, the single nucleotide polymorphism database (dbSNP), 1000 Genomes, as well as online search engines (OMIM and LOVD) were used to search for previously identified variants. Only the mutations previously reported in ataxia telangiectasia patients are classified as deleterious mutations. For the unreported mutations, only nonsense, splice site mutations, frameshift insertions and deletions were considered as deleterious. The pathogenicity of missense mutations were predicted using SIFT [23], PolyPhen2 [24], and MutationTaster [25].

## SUPPLEMENTARY MATERIAL FIGURES AND TABLES


