# Supplementary material for: Prevalence of deleterious ATM germline mutations in gastric cancer patients
Source: Oncotarget. 2015 Oct 23;6(38):40953–8. doi: 10.18632/oncotarget.5944 (PMC4747381; doi:10.18632/oncotarget.5944)
Supplement: Supplementary file 1 [file oncotarget-06-40953-s001.pdf]

Prevalence of deleterious *ATM* germline mutations in gastric cancer patients

Supplementary Material

a. Individual II-7

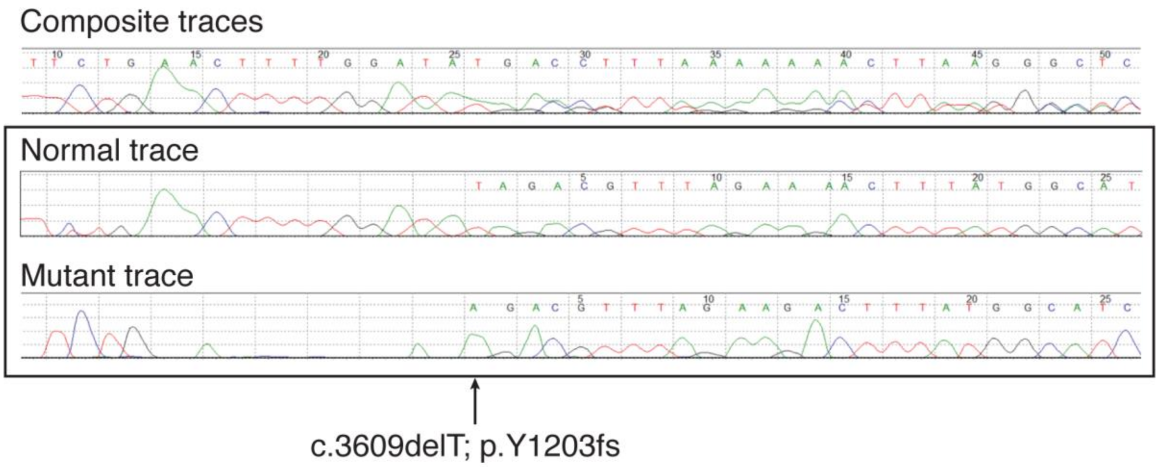

b. Individual II-4

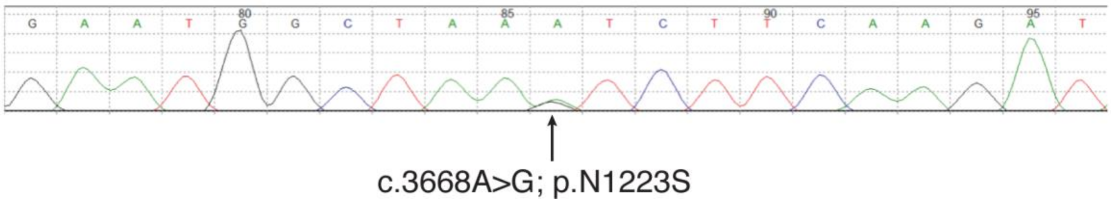

Supplementary Figure 1. Traces of Sanger sequencing for blood DNAs from individual II-7 (a) and individual II-4 (b). The positions of ATM c.3609delT;p.Y1203fs AND c.3668A>AG;p.N1223S were indicated by arrows, separately.

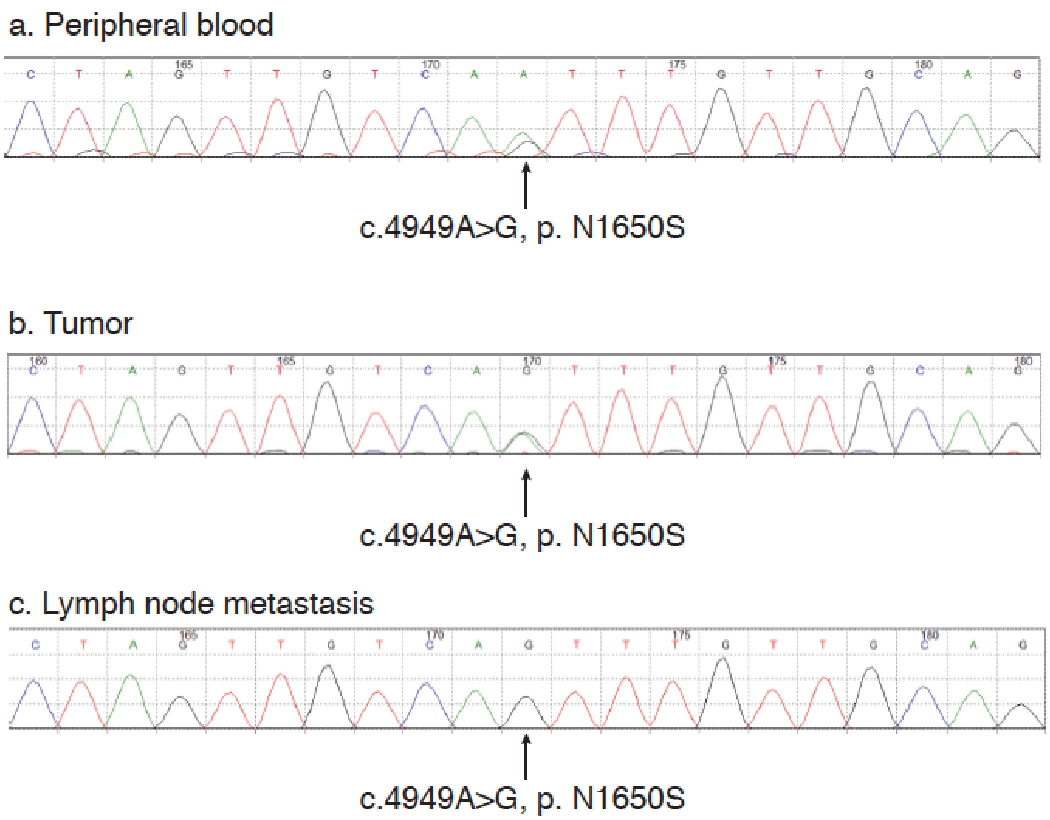

Supplementary Figure 2. Loss of heterozygosity for the *ATM* mutation c.4949A>G;p. N1650S in one patient. Traces of Sanger sequencing were shown for DNAs from a. Peripheral blood; b. Tumor tissue; c. Lymph nodes metastasis.

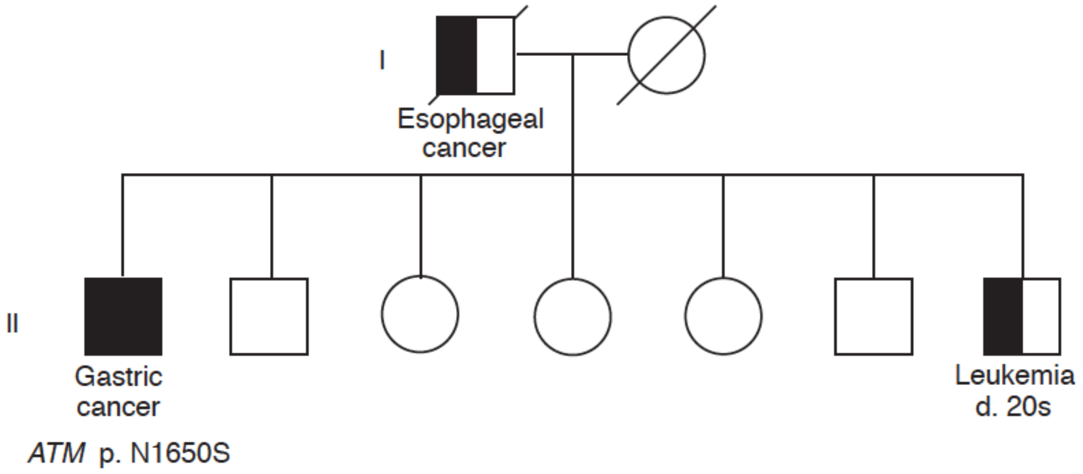

Supplementary Figure 3. Pedigree of the family of the patient with the *ATM* c.4949A>G;p. N1650S germline mutation. The patient with gastric cancer are shaded in black. Half-shaded symbols indicate individuals with non-gastric cancers. Generation I-II are indicated.

Supplementary Table 1. 83 genes used in the custom-designed panel.

ALK	APC	ATM	BARD1	BLM
BMPR1A	BRAF	BRIP1	CASP10	CDH1
CDK4	CDKN2A	CHEK2	DICER1	DPYD
ELAC2	EPCAM	FANCA	FANCB	FANCC
FANCD2	FANCE	FANCF	FANCG	FANCI
FANCL	FANCM	FH	FLCN	KRAS
MAP2K1	MEN1	MET	MITF	MLH1
MRE11A	MSH2	MSH6	MUTYH	NBN
NF1	NF2	NRAS	PALB2	PHOX2B
PMS1	PMS2	PRKAR1A	PTCH1	PTEN
PTPN11	RAD50	RAD51C	RAF1	RB1
RECQL4	RET	RPL11	RPL35A	RPL5
RPS10	RPS17	RPS19	RPS24	RPS26
RPS7	SDHB	SDHC	SDHD	SH2D1A
SMAD4	SOS1	STK11	TP53	TSC1
TSC2	TYMS	VHL	WT1	XIAP
XPA	XPC	ZMAT3		

Supplementary Table 2. Summary of sequencing statistics for individual II-2, II-4, II-5 and II-7 in the Sichuan Chinese family.

Individual	II-2	II-4	II-5	II-7
Read length (bp)	75	75	75	75
Bases mapped to genome	140356920	185843881	197029585	241072641
Bases mapped to targeted region	60770967	49644362	50299432	69462652
Fraction of bases with at least 10 unique reads	94.94%	90.98%	93.36%	94.45%
Effective coverage	107.41	94.19	90.24	124.39
Total number of variants in designed regions	75	80	88	79
Number of variants that are homo-zygous	41	37	36	34
Number of variants that are hetero-zygous	34	43	52	45
Number of variants with MAF>1% in 1000 genome	66	68	75	70
Number of variants in snp database	74	78	85	77

Supplementary Table 3. Predicted pathogenicity of three germline variants in TCGA cohort of 335 gastric cancer patients.

Nucleotide (genomic)	Nucleotide (cDNA)	Amino acid	SIFT prediction	Polyphen2 prediction (HumDiv)	Polyphen2 prediction (HumVar)	MutationTaster prediciton
11:108216616T>G	c.8565T>G	p.S2855R	Damaging	Probably damaging	Probably damaging	Disease Causing
11:108186638G>A	c.6095G>A	p.R2032K	Tolerated	Probably damaging	Possibly damaging	Disease Causing
11:108199929T>G	c.7271T>G	p.V2424G	Damaging	Probably damaging	Probably damaging	Disease Causing

Supplementary Table 4. Deleterious alleles of *ATM* in 1000 genome project data.

Nucleotide (genomic)	Nucleotide (cDNA)	Amino acid	Type	Dbsnp ID	References
11:108121788C>A	c.C1596A	p.C532X	Nonsense	NA	NA
11:108155138C>T	c.C3931T	p.Q1311X	Nonsense	rs200976093	NA
11:108115654C>T	c.C802T	p.Q268X	Nonsense	NA	NA
11:108117828G>T	c.G1039T	p.E347X	Nonsense	NA	NA
11:108142134G>T	c.3077+1G>T		Splicing	NA	NA
11:108170506	c.A5071C	p.S1691R	Missense	rs1800059	Stankovic et al., 1998
